# Identification of Anticancer Target Combinations to Treat Pancreatic Cancer and Its Associated Cachexia Using Constraint-Based Modeling

**DOI:** 10.3390/molecules30153200

**Published:** 2025-07-30

**Authors:** Feng-Sheng Wang, Ching-Kai Wu, Kuang-Tse Huang

**Affiliations:** 1 Department of Chemical Engineering, National Chung Cheng University, Chiayi 621301, Taiwan; kevin88081044@gmail.com; 2 Research Center for Systems Biology and Tissue Engineering, National Chung Cheng University, Chiayi 621301, Taiwan

**Keywords:** constraint-based modeling, cancer metabolism, drug target discovery, biomarker identification, fuzzy optimization, nested hybrid differential evolution

## Abstract

Pancreatic cancer is frequently accompanied by cancer-associated cachexia, a debilitating metabolic syndrome marked by progressive skeletal muscle wasting and systemic metabolic dysfunction. This study presents a systems biology framework to simultaneously identify therapeutic targets for both pancreatic ductal adenocarcinoma (PDAC) and its associated cachexia (PDAC-CX), using cell-specific genome-scale metabolic models (GSMMs). The human metabolic network Recon3D was extended to include protein synthesis, degradation, and recycling pathways for key inflammatory and structural proteins. These enhancements enabled the reconstruction of cell-specific GSMMs for PDAC and PDAC-CX, and their respective healthy counterparts, based on transcriptomic datasets. Medium-independent metabolic biomarkers were identified through Parsimonious Metabolite Flow Variability Analysis and differential expression analysis across five nutritional conditions. A fuzzy multi-objective optimization framework was employed within the anticancer target discovery platform to evaluate cell viability and metabolic deviation as dual criteria for assessing therapeutic efficacy and potential side effects. While single-enzyme targets were found to be context-specific and medium-dependent, eight combinatorial targets demonstrated robust, medium-independent effects in both PDAC and PDAC-CX cells. These include the knockout of SLC29A2, SGMS1, CRLS1, and the RNF20–RNF40 complex, alongside upregulation of CERK and PIKFYVE. The proposed integrative strategy offers novel therapeutic avenues that address both tumor progression and cancer-associated cachexia, with improved specificity and reduced off-target effects, thereby contributing to translational oncology.

## 1. Introduction

Cancer is fundamentally characterized by profound alterations in cellular physiology, among which metabolic dysregulation plays a central role. Tumor cells undergo extensive metabolic rewiring, driven by the activation of oncogenes, inactivation of tumor suppressor genes, and adaptive modifications in intracellular signaling pathways [1,2,3,4]. This reprogramming supports the heightened energetic and biosynthetic demands of malignant proliferation and facilitates survival under dynamic and often hostile microenvironmental conditions, such as hypoxia [3]. The resulting dependence of specific metabolic pathways introduces selective vulnerabilities that can be strategically targeted for therapeutic intervention [5].

Beyond the primary tumor, advanced cancer frequently gives rise to systemic complications such as cachexia—a multifactorial metabolic disorder marked by the progressive loss of skeletal muscle mass, depletion of adipose tissue, and widespread metabolic dysfunction. Cachexia is particularly prevalent in pancreatic cancer (PC) and significantly compromises patient outcomes by reducing treatment tolerance and overall survival [6]. Among the key molecular mediators of cancer cachexia are inflammatory proteins such as Growth Differentiation Factor-15 (GDF-15) and interleukin-6 (IL-6). GDF-15 contributes to muscle atrophy through both extracellular and intracellular mechanisms: it is secreted by tumor cells—often via exosomes—and actives apoptotic signaling cascades within myocytes [7,8]. IL-6, a central cytokine in inflammatory responses, is strongly associated with systemic hyperinflammation in cachexia, and its inhibition has shown therapeutic promise [9,10,11]. Studies have further demonstrated that antibody-mediated inhibition of GDF-15 not only suppresses excessive lipid oxidation, but also aids in maintaining body weight [8]. On a molecular level, GDF-15 exacerbates muscle wasting by activating the Bcl-2/caspase-3 apoptoic pathway and upregulating Atrogin-1, a muscle-specific E3 ubiquitin ligase involved in proteasomal degradation of muscle proteins [12,13,14].

Identifying therapeutic targets that can effectively suppress tumor progression while concurrently alleviating cancer-associated cachexia and minimizing off-target effects remains a major clinical and translational challenge. Cell-specific genome-scale metabolic models (GSMMs) have emerged as powerful computational tools for elucidating the metabolic reprogramming that differentiates malignant cells from their healthy counterparts. By integrating high-throughput omics data—such as transcriptomics, proteomics, and metabolomics—these models enable the simulation and comparative analysis of metabolic flux distributions under diverse physiological and pathological conditions. This systems-level approach allows for the systematic identification of dysregulated metabolic pathways and enzyme-catalyzed reactions that may represent viable intervention points for anticancer therapy.

Despite substantial progress, most previous studies have focused primarily on the intrinsic metabolic alterations of cancer cells [15,16,17], often overlooking the broader metabolic consequences of therapeutic interventions and the development of cancer-associated cachexia. In this study, we employed cell-specific GSMMs to address the following three interrelated dimensions of cancer management: the identification of anticancer targets, the discovery of anti-cachexia targets, and the prediction of drug-induced side effects. To identify anticancer targets, a cell mortality index was incorporated to pinpoint metabolic pathways that are uniquely dysregulated in tumor cells. Potential side effects were anticipated by assessing the metabolic perturbation induced in healthy cells following target-specific intervention. Additionally, anti-cachexia targets were identified by characterizing the degradation reactions of key structural muscle proteins—myosin, actin, and titin—which serve as surrogate biomarkers for muscle wasting. This integrative strategy provides a comprehensive framework for understanding the systemic impact of cancer therapies and supports the development of metabolic interventions that concurrently suppress tumor growth and ameliorate cachexia.

## 2. Results and Discussion

### 2.1. Cell-Specific Genome-Scale Metabolic Models

In this study, the human genome-scale metabolic network (GSMN) Recon3D [18], integrated with lumped reactions representing muscle degradation, was employed as the foundation for reconstructing cell-specific genome-scale metabolic models (GSMMs) to identify potential biomarkers and enzyme targets. Recon3D is a comprehensive human metabolic reconstruction that incorporates metabolic reactions, gene–protein–reaction (GPR) associations, and three-dimensional structural information on enzymes. Building on earlier models such as Recon1 and Recon2, Recon3D substantially expands reaction coverage, featuring over 13,500 metabolic reactions linked to approximately 3300 genes. Notably, it enables systems-level analyses by bridging biochemical function, protein structure, and genetic regulation. As a foundational resource for constraint-based modeling, Recon3D supports a wide array of applications in disease modeling, biomarker discovery, and personalized medicine.

**Figure 1:**
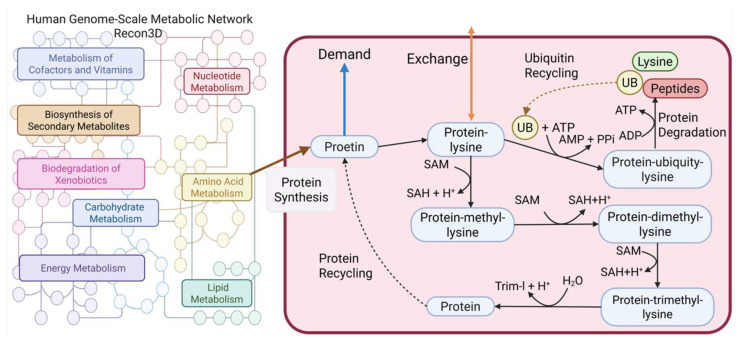
The integration of protein synthesis, degradation, and recycling into the human genome-scale metabolic network (Recon3D) to generate an extended network.

However, Recon3D lacks essential components, including pathways for the synthesis, degradation, and recycling of proinflammatory cytokines (e.g., TNF-α, IFN-γ, IL-6) and key structural proteins such as myosin, actin, and titin. To bridge this gap, a lumped modeling strategy was adopted, wherein protein sequences were used to define the stoichiometry of the missing reactions. These reactions were subsequently integrated into Rencon3D to form an extended GSMN, as illustrated in Figure 1. This extended network served as a generic template, which was then customized using RNA-sequence expression data from cancerous and cachectic tissues to reconstruct cell-specific GSMMs for downstream metabolic analysis and therapeutic design.

RNA-Seq expression data for pancreatic duct adenocarcinoma cancer (PDAC) cells were obtained from the Gene Expression Omnibus (GEO) database of the National Center for Biotechnology Information (NCBI) under accession number GSE183795, based on the study by Yang et al. [19]. This dataset comprises expression profiles from 139 tumor samples and 102 matched adjacent non-tumor tissues, which were used to reconstruct cell-specific genome-scale metabolic models (GSMMs) for both PDAC and its healthy tissue counterparts (HT), with a particular focus on proinflammatory cytokine production. Additionally, gene expression data for cachectic rectus abdominis muscle from PDAC patients were obtained from the study by Narasimhan et al. [20], including 23 cachectic PDAC samples (PDAC-CX) and 11 non-cancer controls. Following the reconstruction protocols described by Cheng et al. [15] and Wang et al. [17], these datasets were used to develop cell-specific GSMMs to characterize muscle degradation involving key structural proteins such as myosin, actin, and titin. The four reconstructed cell-specific GSMMs are provided in Supplementary File S1–S4.

**Figure 2:**
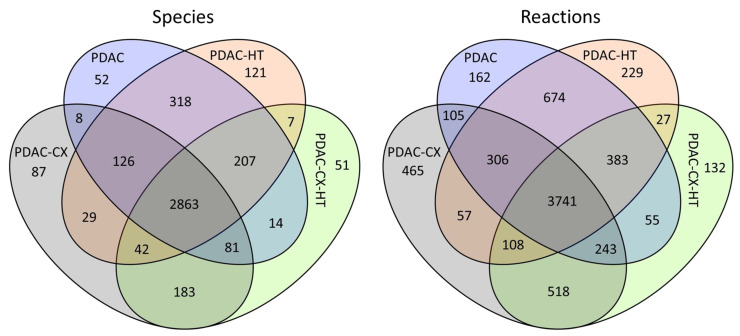
The summary statistics of the reconstructed metabolic models for pancreatic duct adenocarcinoma cancer (PDAC), cachectic PDAC (PDAC-CX), and their corresponding healthy counterparts (PDAC-HT and PDAC-CX-HT).

Figure 2 summarizes the number of metabolites (species) and reactions in these reconstructed models. In this context, a ‘species’ refers to a metabolite assigned to one of nine cellular compartments represented in the metabolic model. The four models had 2863 species and 3741 shared reactions, as shown in the overlapping region in Figure 2. The PDAC model comprised 3669 species and 5669 reactions, while the PDAC-CX model consisted of 3419 species and 5543 reactions. Of these, 52 species and 162 reactions were unique to the PDAC model, whereas 87 species and 465 reactions were specific to the PDAC-CX model. The PDAC network retains approximately 62% of the species and 52.9% of the reactions present in the extended Recon3D model. In comparison, the PDAC-CX network encompasses about 57.8% of the species and 51.8% of the reactions.

### 2.2. Identified Potential Biomarkers

**Figure 3:**
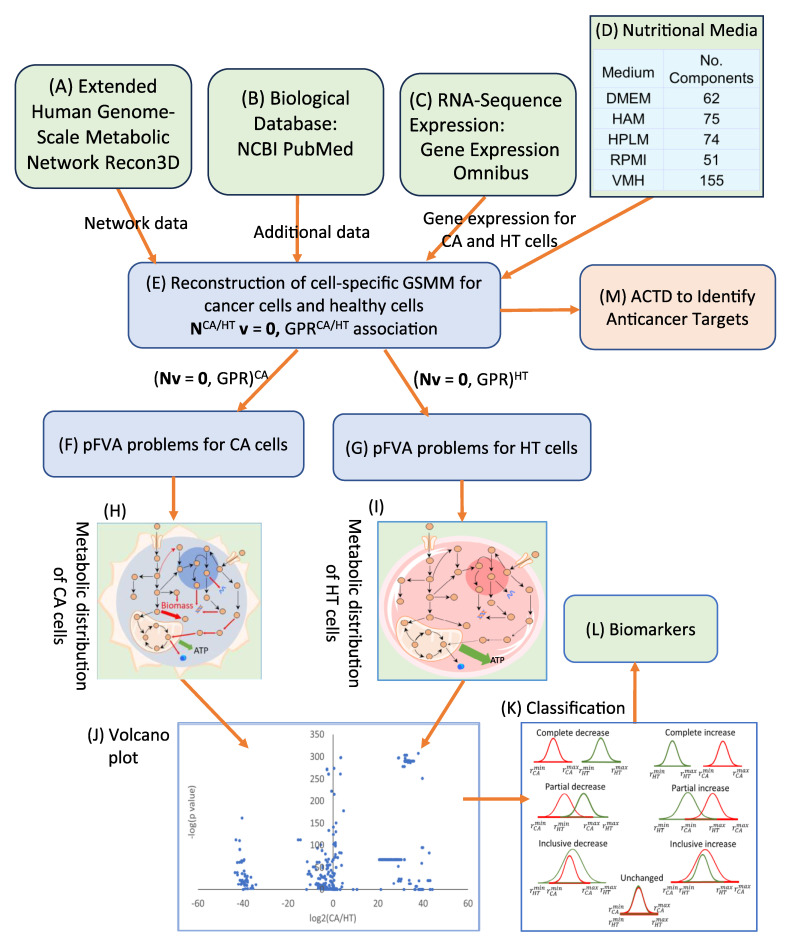
The workflow for biomarker identification using differential expression analysis and Parsimonious Metabolite Flow Variability Analysis.

The identification of potential biomarkers is crucial for inhibiting cancer cell growth and mitigating muscle degradation, particularly in conditions like pancreatic cancer-associated cachexia. To achieve this, a computational framework incorporating Parsimonious Metabolite Flow Variability Analysis (pMFVA) combined with differential expression analysis was employed to identify statistically significant changes in metabolite flow rates (*p*-value < 0.05), as illustrated in Figure 3. This analytical approach was applied across five distinct nutritional media to assess the impact of nutrient availability on biomarker detection. A key aspect of our approach was to identify “medium-independent biomarkers” by focusing on metabolites that exhibited consistent directional changes, showing either a complete increase or a complete decrease across a panel of five distinct nutrient media used in simulations. This rigorous selection process helps to overcome nutrient dependency, ensuring the robustness and broader applicability of the identified biomarkers. Table 1 summarizes the number of detected biomarkers under each condition. In the PDAC model, 524, 507, 483, 536, and 433 biomarkers were identified across the five media, DMEM, HAM, HPLM, RPMI, and VMH, respectively. In contrast, the PDAC-CX model yielded 428, 474, 512, 496, and 504 biomarkers across the same media. These differences highlight the sensitivity of metabolic flux distributions to nutrient availability and suggest the potential for partial or complete overlap in metabolite flow between diseased and healthy states.

**Figure 4:**
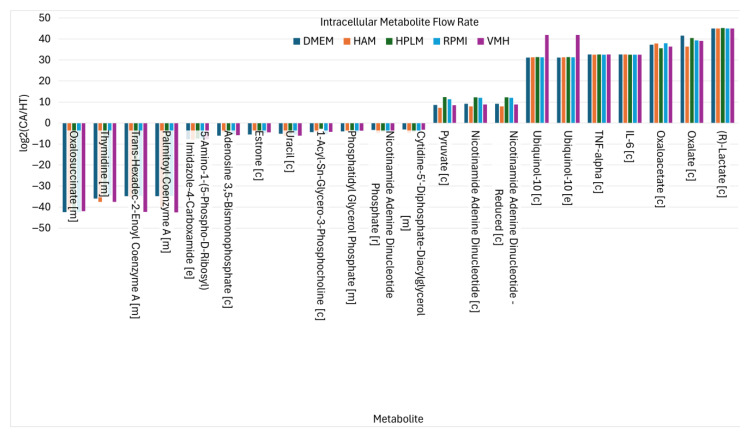
The nutritional medium-independent PDAC biomarkers identified from intracellular metabolite flow rates.

The findings demonstrate that the choice of nutritional medium significantly influences biomarker detection in the PDAC model. To refine biomarker selection, we focused on metabolites that exhibited consistent directional changes, showing either a complete increase or a complete decrease across all five media, as illustrated in Figure 3 (K). This strategy allowed for the identification of medium-independent biomarkers. Figure 4 presents these medium-independent biomarkers, highlighting those with flow rates that consistently increased or decreased across all media.

For example, proinflammatory cytokines such as IL-6 and TNF-α, key components of the proinflammatory biosynthetic pathway, consistently show elevated flow rates in the cancer state, reflecting their established roles in tumor-associated inflammation and pancreatic cancer progression [21,22]. Metabolites involved in glycolysis and redox balance, including pyruvate, NADH, NAD^+^, and lactate, also exhibit consistently increased fluxes. These changes align with the Warburg effect and alter the redox regulation commonly observed in pancreatic cancer cells [23,24]. Ubiquinol-10, located in the inner mitochondrial membrane, facilitates electron transport and ATP production through oxidative phosphorylation. Correspondingly, TCA cycle intermediates such as oxalate and oxaloacetate show sustained increases in flux, supporting enhanced nucleotide and lipid biosynthesis. Additionally, oxaloacetate production via GOT1 contributes to maintaining NAD^+^ regeneration, which is critical for sustaining metabolic and redox homeostasis in cancer cells [21].

**Figure 5:**
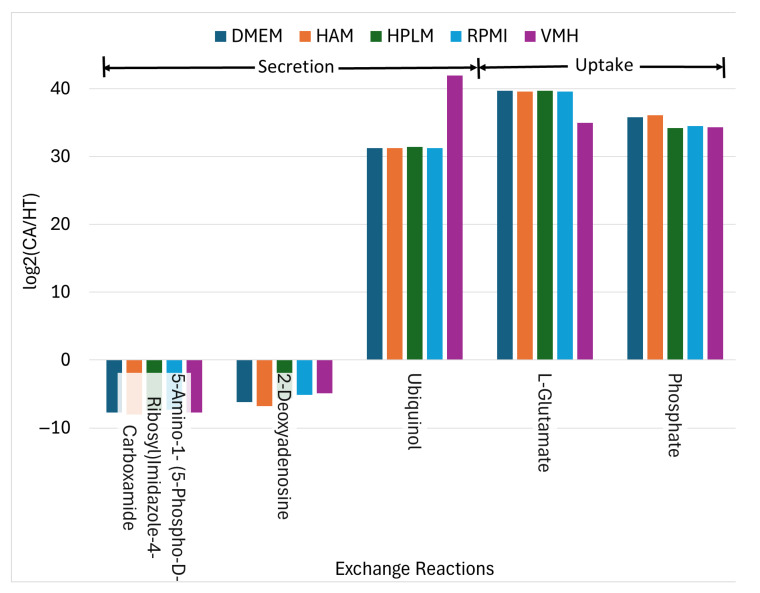
The uptake reactions and secretion reactions identified as nutritional medium-independent PDAC biomarkers.

Conversely, 12 metabolites exhibited consistent decreases in PDAC, indicative of disrupted biosynthesis and compromised NADPH-dependent redox defense. Notably, oxalosuccinate depletion in cancer cells likely results from the diversion of TCA cycle intermediates toward anabolic processes and redox balancing. Fatty acyl-CoA derivatives such as palmitoyl-CoA and trans-hexadec-2-enoyl-CoA also decrease, reflecting altered lipid metabolism. Thymidine levels decline, suggesting perturbations in nucleotide metabolism. Meanwhile, uptake fluxes of phosphate, L-glutamate, and ubiquinol increase consistently, as illustrated in Figure 5, underscoring their importance in supporting cancer cell metabolism. Additionally, the secretion of 2-deoxyadenosine is reduced in cancer cells, which may influence nucleotide salvage pathways [25,26].

**Figure 6:**
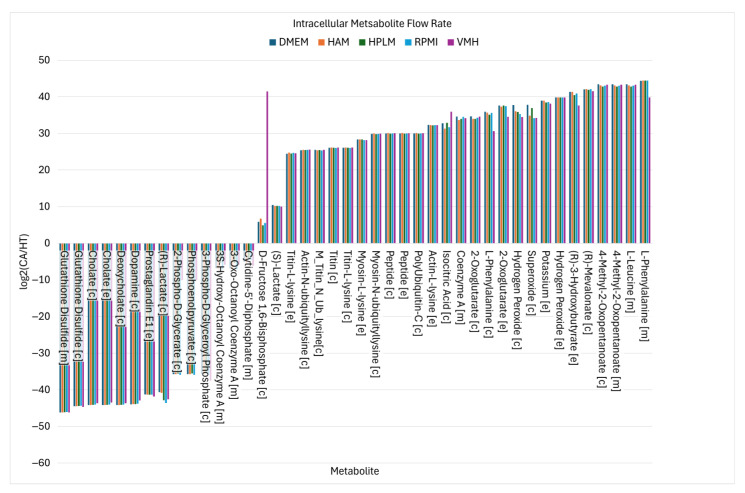
The nutritional medium-independent PDAC-CX biomarkers identified from intracellular metabolite flow rates.

**Figure 7:**
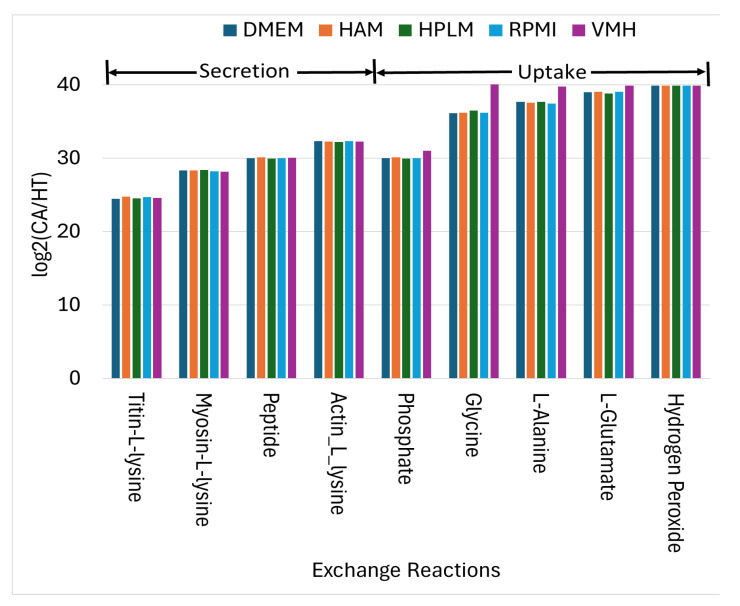
The uptake and secretion reactions identified as nutritional medium-independent PDAC-CX biomarkers.

Using similar analytical procedures, the PDAC-CX model identified a set of medium-independent biomarkers, as shown in Figure 6, comprising 28 metabolites with consistently increased fluxes and 14 metabolites with consistent decreases across all nutritional media. Notably, peptides and degradation products derived from titin, myosin, and actin were markedly elevated, indicating enhanced muscle protein catabolism. These degradation products were subsequently secreted into the extracellular space, as illustrated in Figure 7, aligning with the muscle wasting characteristic of cancer cachexia. Additionally, increased uptake fluxes were observed for L-alanine, glycine, L-glutamate, phosphate, and hydrogen peroxide. Phosphate is critical for the biosynthesis of nucleic acids and membrane phospholipids, and its elevated uptake in proliferative disease states such as cancer supports increased nucleotide synthesis and membrane production, thereby promoting anabolic growth. In contrast, increased uptake of hydrogen peroxide contributes to redox imbalance. While low to moderate levels of hydrogen peroxide act as signaling molecules to promote cellular adaptation and survival, excessive accumulation induces oxidation stress, leading to macromolecular damage and potentially triggering cell death.

### 2.3. Enzyme Targets Predicted Using Constraint-Based Modeling

**Figure 8:**
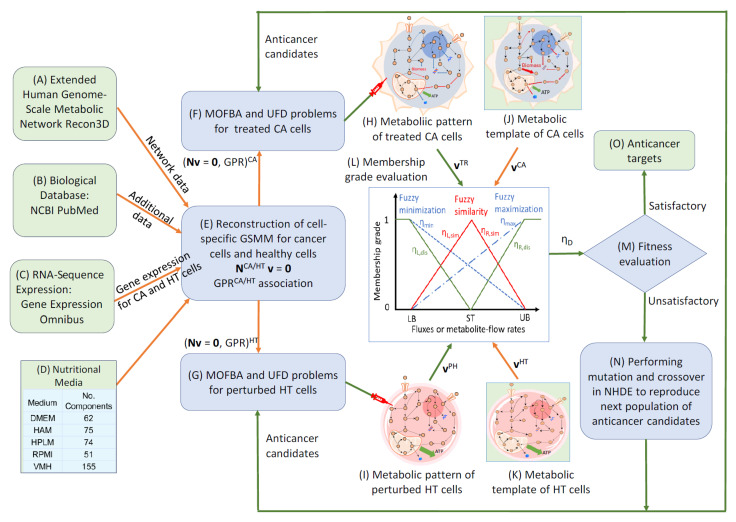
The framework for anticancer target discovery using constraint-based modeling and fuzzy multiobjective optimization. (**A**–**E**) The reconstruction of cell-specific GSMMs and gene–protein–reaction associations, as described in Figure 3. (**F**) The formulation of a Parsimonious Multi-Objective Flux Balance Analysis (pMOFBA) problem for treated CA cells. (**G**) The formulation of a pMOFBA for perturbed HT cells. (**H**) The calculation of metabolic flux distributions in treated CA cells for each anticancer candidate, carried out by solving the pMOFBA model. (**I**) The calculation of metabolic flux distributions in perturbed HT cells for each anticancer candidate via pMOFBA. (**J**) The derivation of the metabolic template of CA cells from clinical data (if available) or baseline pMOFBA without target inregulation. (**K**) The derivation of the metabolic template of HT cells from clinical data (if available) or baseline pMOFBA. (**L**) The transformation of fuzzy multiobjective functions into a fuzzy decision score (*η_D_*) using fuzzy set theory. (**M**) The evaluation of each anticancer candidate’s fitness based on *η_D_* to guide target selection. (**N**) The generation of new anticancer candidates using a nested hybrid differential evolution algorithm if the decision criteria are unmet, with iteration through Figures (**F**) to (**N**). (**O**) The identification of optimal anticancer targets when the decision criterion is satisfied. The lower bound (LB), upper bound (UB), and standard value (ST) are defined by the user, either based on available clinical data or estimated from the metabolic templates of cancer (CA) and healthy (HT) cells.

The anticancer target discovery (ACTD) platform proposed by Wang and Zhang [17], illustrated in Figure 8, was employed to identify enzyme targets for treating PDAC and PDAC-CX across five distinct nutritional media. The predicted targets are detailed in Supplementary File S1. In this framework, cell viability (CV) and metabolic deviation (MD) were used as dual decision-making criteria to evaluate the therapeutic efficacy of each enzyme target. For PDAC, five objective functions were simultaneously minimized to determine CV, with the membership grade ranging from 0 and 1. The highest priority was assigned to abolishing biomass production, while additional objectives included the reduction of ATP synthesis and proinflammatory cytokine production (IL6, IFN-γ, and TNF-α).

For PDAC-CX, the primary objective was to minimize myosin degradation, followed by reductions in actin and titin degradation―reflecting muscle preservation goals in cachexia management. Each candidate target was further evaluated for its impact on healthy tissue (denoted as PB, perturbed by treatment) by examining alterations in flux distribution. The MD metric―normalized between 0 and 1 using a linear membership function―quantified the similarity between PB and healthy tissue (HT), as well as the dissimilarity between PB and the corresponding diseased models. This metric served as a proxy for predicting the potential side effects associated with each enzyme target.

**Figure 9:**
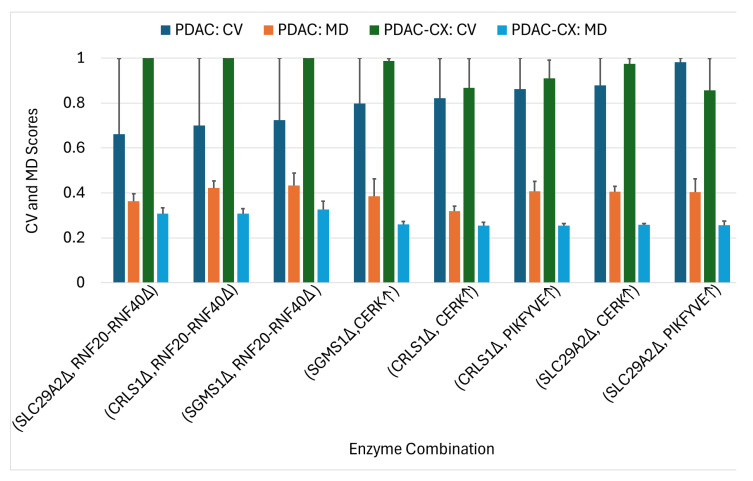
The average cell viability (CV) and metabolic deviation (MD) of medium-independent target combinations for the treatment of PDAC and PDAC-CX cells. The symbols Δ and ↑ denote knockout and upregulation, respectively.

Using the ACTD platform, 27 single-enzyme targets for PDAC treatment were identified across five distinct nutritional media, as detailed in Supplementary File S5. Several targets resulted in zero cell viability (CV) in certain media, indicating complete treatment failure under those specific conditions; however, other targets were able to inhibit cancer growth. These findings suggest that the efficacy of the identified targets is dependent on the nutritional environment. Notably, none of these single-enzyme targets were effective against the PDAC-CX model. Conversely, 30 single-enzyme targets identified for PDAC-CX treatment failed to produce therapeutic effects in PDAC, highlighting the context-specific nature of these interventions. To address this limitation, the ACTD platform was extended to a combinatorial targeting strategy aimed at identifying enzyme target pairs that are effective against both PDAC and PDAC-CX. Some of these combinations, also listed in Supplementary File S5, yielded zero CV but remained sensitive to medium conditions. Among them, seven target combinations emerged as robust candidates exhibiting medium-independent efficacy. The average values of cell viability (CV) and metabolic deviation (MD) for these medium-independent combinations when treating both PDAC and PDAC-CX are summarized in Figure 9.

The results reveal that the identified combinations comprise eight enzyme targets, including five individually encoded enzymes and one enzyme complex―namely the RNF20–RNF40 complex. Among these, four enzymes (SLC29A2, RNF20–RNF40, CRLS1, and SGMS1) are knocked out, while two enzymes (CERK and PIKFYVE) are upregulated to achieve therapeutic efficacy. In the PDAC model, the average cell viability (CV) ranged from 0.66 to 0.98, and the metabolic deviation (MD) from 0.32 to 0.43. In the PDAC-CX model, the CV ranged from 0.86 to 1.0 and the MD from 0.25 to 0.33. Higher values of CV and MD reflect increased therapeutic efficacy and reduced potential side effects. Collectively, the eight identified target combinations represent effective therapeutic strategies for concurrently treating both PDAC and PDAC-CX cells. Furthermore, the CV and MD metrics provide a quantitative basis for selecting the optimal trade-off between efficacy and safety.

SLC29A2 (solute carrier family 29 member 2), also known as ENT2, is a bidirectional transporter that regulates intracellular homeostasis by mediating nucleosides and nucleobase uptake and efflux. Its knockout disrupts nucleotide salvage pathways, impairs cellular energy balance, and inhibits cancer cell proliferation. In the PDAC-CX model, the RNF20–RNF40 complex functions as a key E3 ubiquitin ligase responsible for ubiquitinating protein-L-lysine residues―including those in myosin, actin, and titin. Knockout of this complex may impair lysine and ubiquitin recycling, thereby promoting enhanced protein degradation (Figure 1). Consequently, dual targeting of SLC29A2 and the RNF20–RNF40 complex represents a promising therapeutic strategy for PDAC and PDAC-CX cells.

Knockout of the **SGMS1** (*sphingomyelin synthase 1*) gene leads to significant metabolic disruption by impairing sphingolipid metabolism, thereby reducing cancer cell viability and inhibiting tumor progression. SGMS1 catalyzes the synthesis of sphingomyelin by transferring a phosphocholine group from phosphatidylcholine to N-acylsphingosine. Similarly, knockout of the **CRLS1** (*cardiolipin synthase 1*) gene impairs mitochondrial lipid metabolism by blocking cardiolipin biosynthesis, which involves the conversion of cytidine-5′-diphosphate-diacylglycerol and phosphatidylglycerol. Both sphingomyelin and cardiolipin are critical lipid components essential for biomass synthesis in the PDAC model. Consequently, the knockout of either **SGMS1** or **CRLS1** abolishes biomass production. Therefore, co-targeting **SGMS1** or **CRLS1** in combination with the **RNF20-RNF40** complex constitutes a promising therapeutic strategy for the simultaneous treatment of PDAC and PDAC-CX cells.

Upregulation of CERK (ceramide kinase) or PIKFYVE (phosphoinositide kinase) alone is insufficient to prevent muscle degradation in the PDAC-CX model across all five nutritional media, as shown in Supplementary File S5. CERK catalyzes the phosphorylation ceramide to generate ceramide-1-phosphate (C1P). Deletion or downregulation of CERK results in ceramide accumulation, promoting pro-catabolic and pro-apoptotic signaling in muscle cells. This effect is particularly detrimental in PDAC-associated cachexia, where inflammation, energy imbalance, and lipid metabolism are already dysregulated. Although CERK upregulation may exert protective effects by shifting sphingolipid balance toward C1P, the current dataset provides no direct evidence supporting this outcome. Nevertheless, previous studies have associated CERK deficiency with muscle atrophy, underscoring its therapeutic potential for preserving muscle mass under cachectic conditions [27,28,29].

Similarly, upregulation of PIKFYVE may modulate muscle metabolism through its involvement in insulin sensitivity and glucose uptake. While most existing studies emphasize the detrimental effects of PIKFYVE deficiency―such as impaired insulin signaling and glucose homeostasis [30]―its upregulation could potentially enhance these metabolic pathways, thereby contributing to muscle preservation. However, direct evidence supporting this effect remains limited. Consequently, combining the upregulation of CERK or PIKFYVE with the knockout of SLC29A2, SGMS1, or CRLS1 may constitute a more effective therapeutic strategy for concurrently targeting both PDAC and PDAC-CX cells across all five media conditions, as illustrated in Figure 9.

## 3. Methods

### 3.1. Extension of Human Genome-Scale Metabolic Network

Figure 1 illustrates the workflow used to reconstruct cell-specific genome-scale metabolic networks (GSMNs) for PDAC and PDAC-CX cells, based on transcriptomic data. The RNA-Seq data for PDAC cells were obtained from the Gene Expression Omnibus (GEO) database at the National Center for Biotechnology Information (accession number GSE183795), as reported by Yang et al. [19]. The transcriptomic data for PDAC-CX cells were sourced from the study by Narasimhan et al. [20]. These datasets were processed using established reconstruction protocols described in previous studies [15,16,17] to generate the respective cell-specific GSMNs. However, the standard human genome-scale metabolic network, Recon3D [18], lacks essential pathways for the synthesis, degradation, and recycling of key proinflammatory cytokines (e.g., TNF-α, IFN-γ, IL-6) and structural proteins (e.g., myosin, actin, and titin). To address this limitation, a lumped-reaction approach was applied, wherein protein sequences were used to define and construct the missing reactions. These reactions were subsequently incorporated into Rencon3D to create an extended GSMN, as depicted in Figure 1.

Each proinflammatory cytokine or skeletal muscle protein is modeled as a polymerization reaction based on its amino acid sequence, retrieved from the UniProt database (https://www.uniprot.org/ (accessed on 20 June 2024)). The generalized mass balance for protein polymerization is expressed as follows:

(1) $$\sum_{A_{j}}S_{A_{j}}A_{j}+S_{H_{2}O}H_{2}O+S_{ATP}ATP\to S_{Protein}\langle \sum_{j}\rangle A_{j}+S_{ADP}ADP+S_{P_{i}}P_{i}+S_{H^{+}}H^{+}$$

Here, $\langle \sum_{j}A_{j}\rangle$ denotes the synthesized protein composed of amino acids $A_{j}$. The reaction involves water $\left(H_{2}O\right)$, adenosine triphosphate $\left(ATP\right)$, adenosine diphosphate $\left(ADP\right)$, inorganic phosphate $\left(P_{i}\right)$, and proton $\left(H^{+}\right)$. The stoichiometric coefficients (in mmole per gram of protein) are calculated as follows:

(2) $$S_{A_{j}}=\frac{10^{3}M_{A_{j}}}{M_{Protein}}$$

(3) $$S_{H_{2}O}=\frac{10^{3}\left(k_{ATP}-1\right)\left(\sum_{A_{j}}M_{A_{j}}-1\right)}{M_{Protein}}$$

(4) $$S_{ATP}=S_{ADP}=S_{P_{i}}=S_{H_{2}O}=\frac{10^{3}k_{ATP}\left(\sum_{A_{j}}M_{A_{j}}-1\right)}{M_{Protein}}$$

(5) $$S_{ATP}=S_{ADP}=S_{P_{i}}=S_{H_{2}O}=\frac{10^{3}k_{ATP}\left(\sum_{A_{j}}M_{A_{j}}-1\right)}{M_{Protein}}$$

(6) $$S_{Protein}=\frac{1000\;}{M_{Protein}}$$

In these equations, $M_{protein}$ is the molecular weight of the synthesized protein, $M_{A_{j}}$ is the molar mass of each amino acid $A_{j}$, and $k_{ATP}$ denotes the number of *ATP* molecules required per peptide bond, typically ranging from 4 to 4.3 [31]. Detailed amino acid compositions and molar contributions are provided in references [32,33].

Finally, the polymerization and degradation reactions, along with amino acid recycling pathways for each cytokine and structural protein, were incorporated into the extended Recon3D model. Using this augmented network, cell-specific GSMNs for PDAC and PDAC-CX were reconstructed following the established procedures described in references [15,16,17].

### 3.2. Parsimonious Multi-Objective Flux Balance Analysis

Parsimonious Multi-Objective Flux Balance Analysis (pMOFBA) improves upon classical flux balance analysis by introducing an additional optimization criterion: the minimization of the total fluxes across the metabolic network. This method is formulated as a two-stage optimization framework that integrates Multi-Objective Flux Balance Analysis (MOFBA) with a Uniform Flux Distribution (UFD) strategy:

(7) $$\begin{array}{cc} 1^{st}\;stage:MOFBA\; & 2^{nd}\;stage:UFD\;problem\\ \left\{\begin{array}{c} \begin{array}{c} \underset{v_{f},v_{b}}{\max}z_{1}=v_{biomass}\\ \begin{array}{c} \underset{v_{f},v_{b}}{\max}z_{2}=v_{ATP}\\ \underset{v_{f},v_{b}}{\max}z_{P_{k}}=v_{P_{k}},\forall k\in \left\{proteins\right\} \end{array}\\ \begin{array}{c} subject\;to\;\\ N\left(v_{f}-v_{b}\right)=0\\ \begin{array}{c} 0\leq v_{f}^{LB}\leq v_{f}\leq v_{f}^{UB}\;\\ 0\leq v_{b}^{LB}\leq v_{b}\leq v_{b}^{UB}\; \end{array} \end{array} \end{array}\\ GPR\;association\; \end{array}\right. & \left\{\begin{array}{c} \underset{v_{f},v_{b}}{\min}\sum_{i=1}^{n}w_{i}\left(v_{f,i}+v_{b,i}\right)\\ subject\;to\;\\ \begin{array}{c} N\left(v_{f}-v_{b}\right)=0\\ 0\leq v_{f}^{LB}\leq v_{f}\leq v_{f}^{UB}\;\\ \begin{array}{c} 0\leq v_{b}^{LB}\leq v_{b}\leq v_{b}^{UB}\;\\ GPR\;association\;\\ z_{1}\geq z_{1}^{*},z_{2}\leq z_{2}^{*},\;z_{P_{i}}\leq z_{P_{i}}^{*}\; \end{array} \end{array} \end{array}\right. \end{array}$$

In this framework, the first stage (MOFBA) determines an optimal flux distribution $\left(v_{f}^{*},v_{b}^{*}\right)$ that simultaneously maximizes multiple biologically relevant objectives, such as biomass production $\left(z_{1}\right)$, ATP generation $\left(z_{2}\right)$, and related-protein synthesis $\left(z_{P_{k}}\right)$. The second stage (UFD) then minimizes the total weighted flux through the network to enforce a parsimonious (biologically efficient) flux profile while ensuring that the performance levels obtained in the first stage $\left(z_{1}^{*},z_{2}^{*},z_{P_{k}}^{*}\right)$ are maintained.

Here, $v_{f}$ and $v_{b}\;$represent the forward and backward flux vectors, respectively. The stoichiometric matrix $N\in {\mathbb{R}}^{m\times n}$ defines the metabolic network, where m is the number of metabolites and *n* the number of reactions. Fluxes are constrained by lower $\left(v_{f}^{LB}\;and\;v_{b}^{LB}\right)$ and upper $\left(v_{f}^{UB}\;and\;v_{b}^{UB}\right)$ bounds. Gene–protein–reaction (GPR) associations impose regulatory constraints based on gene expression and enzyme availability, enabling integration with transcriptomic data. These constraints are consistently applied to both diseased-specific GSMNs and their corresponding healthy models.

In the UFD model, the weights $w_{i}\;$reflect the level of transcriptomic confidence associated with each reaction and are assigned based on a quartile-based classification scheme:

(8) $$w_{i}=\left\{\begin{array}{c} 1/4,\;i\in \left\{high\;confidence\;reactions\right\}\;\\ 1/2,i\in \left\{medium\;confidence\;reactions\right\}\;\\ \begin{array}{c} 3/4,i\in \left\{negative\;confidence\;reactions\right\}\;\\ 1,i\in \left\{others\;or\;non-gene-association\;reaction\right\} \end{array} \end{array}\right.$$

This weighting scheme biases the solution toward flux distributions that prioritize reactions with strong transcriptomic support, thereby promoting biologically meaningful and resource-efficient metabolic activity. Consequently, pMOFAB improves both the interpretability and predictive accuracy of reconstructed metabolic models by aligning flux predictions with gene expression profiles and cellular resource constraints.

### 3.3. Parsimonious Metabolite Flow Variability Analysis

Parsimonious Metabolite Flow Variability Analysis (pMFVA), which incorporates multi-objective functions, is formulated as follows:

(9) $$\left\{\begin{array}{c} \underset{v_{f},v_{b},\varsigma \in [0,1]}{\max/\min}r_{m_{k}},\;m_{k}\in \Omega^{m}\\ \begin{array}{cc} 1^{st}\;stage:MOFBA & 2^{nd}\;stage:UFD\;problem\\ \left\{\begin{array}{c} \begin{array}{c} \underset{v_{f},v_{b}}{\max}z_{1}=v_{biomass}\\ \begin{array}{c} \underset{v_{f},v_{b}}{\max}z_{2}=v_{ATP}\\ \underset{v_{f},v_{b}}{\max}z_{P_{k}}=v_{P_{k}},\forall k\in \left\{proteins\right\} \end{array}\\ \begin{array}{c} subject\;to\;\\ N\left(v_{f}-v_{b}\right)=0\\ \begin{array}{c} 0\leq v_{f}^{LB}\leq v_{f}\leq v_{f}^{UB}\;\\ 0\leq v_{b}^{LB}\leq v_{b}\leq v_{b}^{UB}\; \end{array} \end{array} \end{array}\\ GPR\;association\; \end{array}\right. & \left\{\begin{array}{c} \underset{v_{f},v_{b}}{\min}\sum_{i=1}^{n}w_{i}\left(v_{f,i}+v_{b,i}\right)\\ subject\;to\;\\ \begin{array}{c} N\left(v_{f}-v_{b}\right)=0\\ 0\leq v_{f}^{LB}\leq v_{f}\leq v_{f}^{UB}\;\\ \begin{array}{c} 0\leq v_{b}^{LB}\leq v_{b}\leq v_{b}^{UB}\;\\ GPR\;association\;\\ z_{1}\geq {\zeta z}_{1}^{*},z_{2}\leq {\zeta z}_{2}^{*},\;z_{P_{i}}\leq {\zeta z}_{P_{i}}^{*} \end{array} \end{array} \end{array}\right. \end{array} \end{array}\right.$$

Here, $\left(z_{1}^{*},z_{2}^{*},z_{P_{k}}^{*}\right)\;$denote the optimal objective values obtained through the MOFBA solution at ς=1, representing the fully optimal metabolic state. The parameter $\varsigma \in \left[0,1\right]$ serves as an outer-level decision variable in the pMFVA framework, enabling evaluation under optimal $\left(\varsigma =1\right)$ and suboptimal $\left(\varsigma <1\right)$ conditions. The goal of pMFVA is to determine the maximum and minimum feasible flow rates of key metabolites $r_{m_{k}}$ across the entire range of ς, while also generating a set of flux distributions associated with varying levels of metabolic performance. This facilitates a comprehensive assessment of metabolic flexibility and robustness under different physiological or perturbation scenarios.

After solving the MOFBA problem, the UFD problem is iteratively solved for various values of $\varsigma \in \left[0,1\right)$, generating a spectrum of metabolite flow rates that is defined as follows:

(10) $$r_{m_{k}}\left(\varsigma \right)=\sum_{N_{ij>0,j=1}}^{n}N_{ij}v_{f.j}^{*}\left(\varsigma \right)-\sum_{N_{ij}<0,j=1}^{n}N_{ij}v_{b,j}^{*}\left(\varsigma \right),\;m_{k}\in \Omega^{m},\;\varsigma \in \left[0,1\right]$$

The overall variability range for each metabolite flow is then computed as follows:

(11) $$\left[r_{m_{k}}^{max},\;r_{m_{k}}^{min}\;\right]=\left[\underset{\varsigma }{\min}\left\{r_{m_{k}}\left(\varsigma \right)\right\},\underset{\varsigma }{\max}\left\{r_{m_{k}}\left(\varsigma \right)\right\}\right]$$

This framework enables a biologically meaningful and parsimonious characterization of metabolite flow variability. By applying Equations (9) and (10), two sets of metabolite flow rates―corresponding to diseased and healthy conditions―can be obtained. Comparative analysis of these profiles allows for the identification of differential expressed metabolic flows, which may serve as potential biomarkers, as illustrated in Figure 3.

### 3.4. Anticancer Target Discovery Framework

**Figure 10:**
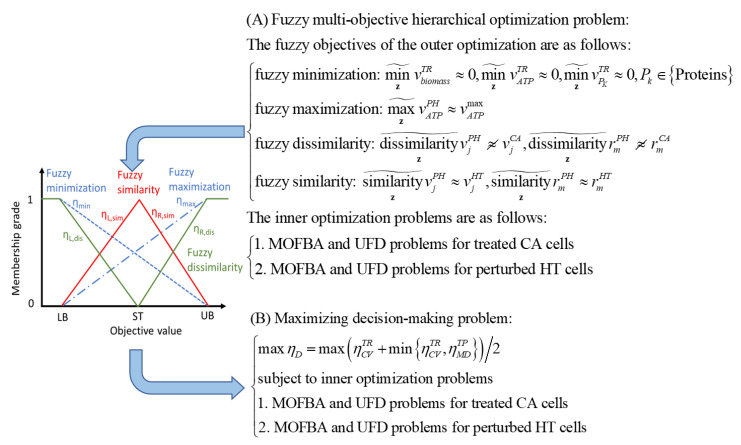
The transformation of a fuzzy multi-objective hierarchical optimization problem into a maximizing decision-making problem using fuzzy membership functions. The lower bound (LB), upper bound (UB), and standard value (ST) are defined by the user, either based on available clinical data or estimated from the metabolic templates of cancer (CA) and healthy (HT) cells.

Figure 8 represents the anticancer target discovery (ACTD) framework, as proposed by Wang and Zhang [17]. This framework is formulated as a fuzzy multi-objective hierarchical optimization problem designed to identify potential therapeutic targets for the simultaneous treatment of PDAC and PDAC-CX, as illustrated in Figure 10. As shown in Figure 10, fuzzy minimization and maximization objectives are used to evaluate treatment-associated cell mortality in diseased cells and cell survival in healthy cells. These objectives are assessed using one-sided linear membership functions, represented by dashed and dot-dashed lines, respectively. The resulting membership values are used to define cell viability, denoted as $\eta_{CV}\in \left[0,1\right]$, which quantifies the therapeutic effect in terms of efficacy and selectivity.

Healthy cells are perturbed by the application of a therapeutic target, leading to altered flux distributions. These perturbed flux states—referred to as PH (perturbed healthy) cells—are used to evaluate metabolic deviations in comparison to both cancer (CA) cells and unperturbed healthy tissue (HT) cells. To quantify these deviations, fuzzy dissimilarity and similarity measures are applied to assess flux differences. Two-sided linear membership functions are used for this purpose, with green lines representing fuzzy dissimilarity and red lines representing fuzzy similarity. These functions are used to define metabolic deviation, denoted as $\eta_{MD}\in \left[0,1\right]$, which serves as an indicator for estimating the potential side effects of a given target. Based on these membership function definitions, the fuzzy multi-objective hierarchical optimization problem is reformulated as a maximizing decision-making problem. This problem is then solved using the nested hybrid differential evolution to identify candidate therapeutic targets that balance efficacy and safety.

## 4. Conclusions

This study presents an integrative systems biology framework for the identification of therapeutic targets capable of simultaneously treating pancreatic ductal adenocarcinoma (PDAC) and its associated cachexia (PDAC-CX). By extending the Recon3D network to incorporate pathways for protein synthesis and degradation, and by constructing cell-specific genome-scale metabolic models informed by transcriptomic data, the framework effectively captures the critical metabolic alterations underlying both tumor progression and muscle wasting.

The application of Parsimonious Metabolite Flow Variability Analysis across multiple nutritional environments enabled the identification of robust, medium-independent biomarkers. These biomarkers revealed key aspects of metabolic reprogramming in both PDAC and cachectic muscle cells, including disruptions in redox homeostasis, lipid metabolism, nucleotide biosynthesis, and inflammatory signaling pathways.

Using the anticancer target discovery platform, enzyme targets were systematically evaluated through a fuzzy multi-objective optimization framework. While single-enzyme interventions exhibited context-specific and nutrient-dependent effects, combinatorial targeting strategies revealed eight enzyme target pairs with consistent, medium-independent efficacy. Notably, combinations involving knockouts of SLC29A2, SGMS1, CRLS1, and the RNF20–RNF40 complex, alongside the upregulation of CERK and PIKFYVE, demonstrated strong therapeutic potential by achieving favorable trade-offs between efficacy (cell viability) and safety (metabolic deviation).

This study introduces a novel computational framework for dual-target discovery in cancer and cachexia, facilitating the rational design of metabolism-based therapies with enhanced clinical relevance. The findings highlight the necessity of accounting for systemic effects and nutrient variability during anticancer drug development, thereby advancing translation strategies aimed at simultaneously suppressing tumor growth and alleviating cancer-associated muscle wasting.

Despite these promising insights, it is important to recognize that all findings to date are based solely on computational modeling and transcriptomic data integration. Consequently, the predicted enzyme targets—particularly combinatorial candidates such as SLC29A2, SGMS1, CRLS1, CERK, PIKFYVE, and the RNF20–RNF40 complex—require rigorous experimental validation to confirm their biological relevance and therapeutic potential. Future investigations should prioritize both in vitro and in vivo studies, utilizing CRISPR-based gene editing or targeted pharmacological inhibition in appropriate PDAC and cachexia models. Furthermore, leveraging clinical datasets and patient-derived organoids offers a translational platform to assess the safety and efficacy of these interventions in physiologically relevant systems. Collectively, these approaches are critical for translating in silico predictions into clinically actionable strategies and ultimately advancing the development of more effective and personalized therapies for PDAC and its associated systemic complications.

## Figures and Tables

**Table 1:** The number of identified biomarkers in PDAC and PDAC-CX based on the metabolite flow rates across five nutritional media. Abbreviations: CI―complete increase; PI―partial increase; II―inclusive increase; ID―inclusive decrease; PD―partial decrease; CD―complete decrease (as defined in Figure 3 (K)).

Medium	Type	CI	PI	II	ID	PD	CD	Total
DMEM	PDAC	97	240	12	26	4	145	524
	PDAC-CX	80	134	16	58	24	116	428
HAM	PDAC	132	212	12	20	6	125	507
	PDAC-CX	78	130	20	72	20	154	474
HPLM	PDAC	125	211	17	32	7	91	483
	PDAC-CX	88	175	17	112	26	94	512
RPMI	PDAC	134	222	11	15	6	148	536
	PDAC-CX	95	146	20	89	21	125	496
VMH	PDAC	63	203	10	31	8	118	433
	PDAC-CX	140	171	25	43	29	96	504

## Data Availability

The source programs of the anticancer target discovery platform and the cell-specific genome-scale metabolic models were coded by the General Algebraic Modeling System (GAMS, https://www.gams.com/ (accessed on 20 June 2024)) and are available at http://doi.org/10.5281/zenodo.10771499 (accessed on 20 June 2024). The data of this study are available in the Gene Expression Omnibus database (https://www.ncbi.nlm.nih.gov/geo/query/acc.cgi?acc=GSE183795) accessed on 20 June 2024 and (https://www.ncbi.nlm.nih.gov/geo/query/acc.cgi?acc=GSE133979), accessed on 20 June 2024.

## Supplementary Materials

The following supporting information can be downloaded at: https://www.mdpi.com/article/10.3390/molecules30153200/s1, Supplementary File S1. Reconstruction of cell-specific genome-scale metabolic network for pancreatic ductal adenocarcinoma (PDAC). Supplementary File S2. Reconstruction of cell-specific genome-scale metabolic network for healthy pancreatic tissue (PDAC-HT). Supplementary File S3. Reconstruction of cell-specific genome-scale metabolic network for PDAC associated cachexia (PDAC-CX). Supplementary File S4. Reconstruction of cell-specific genome-scale metabolic network for healthy tissue associated with PDAC-CX (PDAC-CX-HT). Supplementary File S5. Identified single-enzyme targets for PDAC and PDAC-CX, and combinatorial targets for simultaneously treating both conditions.
